# The patient pathway for mild cognitive impairment due to Alzheimer’s disease in Asia: Current practices, barriers, and expert recommendations for optimization

**DOI:** 10.1016/j.tjpad.2025.100215

**Published:** 2025-06-06

**Authors:** Seong Hye Choi, SangYun Kim, Paulus Anam Ong, Ai Vyrn Chin, Jacqueline Dominguez, Christopher Li-Hsian Chen, Vorapun Senanarong, Chaur-Jong Hu, Manjari Tripathi, Vincent Mok, Gandan Jiang, Amitabh Dash

**Affiliations:** a Department of Neurology, College of Medicine, Inha University, Incheon, South Korea; b Department of Neurology, Seoul National University College of Medicine and Seoul National University Bundang Hospital, Seoul, South Korea; c Department of Neurology, Hasan Sadikin Hospital, Universitas Padjadjaran, Bandung, Indonesia; d The Ageing and Age-Associated Diseases Research Group and Unit of Geriatric Medicine, Faculty of Medicine, University Malaya Medical Centre, Kuala Lumpur, Malaysia; e Institute of Neurosciences, St. Luke’s Medical Center, Quezon City, Philippines; f Memory, Ageing and Cognition Centre, Departments of Pharmacology and Psychological Medicine, Yong Loo Lin School of Medicine, National University of Singapore, Singapore; g Department of Medicine, Faculty of Medicine, Siriraj Hospital, Mahidol University, Bangkok, Thailand; h Department of Neurology, College of Medicine, Taipei Medical University, Taipei, Taiwan; i Department of Neurology, All India Institute of Medical Sciences, New Delhi, India; j Division of Neurology, Department of Medicine and Therapeutics, Faculty of Medicine, The Chinese University of Hong Kong, Hong Kong, China; k Eisai Co., Ltd, Tokyo, Japan; l Eisai Singapore Pte. Ltd., Singapore

**Keywords:** Mild cognitive impairment, Alzheimer’s disease, Patient journey, Disease-modifying treatment, Asia

## Abstract

**Background:**

The age-standardized prevalence of Alzheimer’s disease in Asia has increased rapidly in recent years. Disease-modifying treatments that can slow disease progression are now becoming available for patients with early-stage Alzheimer’s disease, including those with mild cognitive impairment. However, challenges in diagnosis and assessment for these patients remain.

**Objectives:**

This study characterized the care pathway for mild cognitive impairment due to Alzheimer’s disease in Asia, including barriers to care, and considered the future treatment landscape, with the aim of making recommendations for optimizing the care pathway in readiness for the availability of new disease-modifying treatments.

**Design:**

Qualitative study based on semi-structured interviews.

**Setting:**

Interviews were conducted with physicians in general/tertiary hospitals in Hong Kong, India, Indonesia, Korea, Malaysia, the Philippines, Singapore, Taiwan, and Thailand. Physicians from mainland China and Japan were not included.

**Participants:**

Physicians managing patients with mild cognitive impairment.

**Measurements:**

Number and/or proportion of participants providing a given response, and numerical estimates provided by interview participants.

**Results:**

Forty-four physicians, primarily neurologists (*n* = 31; 70.5 %), were interviewed. Participants managed a median of 67.5 patients with mild cognitive impairment per month, of whom 24.0–87.5 % had mild cognitive impairment due to Alzheimer’s disease. Clinical investigations routinely comprised brief neuropsychological assessments, such as the Mini-Mental State Examination (*n* = 41), as well as neurological tests (*n* = 39) and magnetic resonance imaging (*n* = 40). Except in Korea, comprehensive neuropsychological test batteries and amyloid positron emission tomography were seldom conducted in Asia. Most patients with mild cognitive impairment due to Alzheimer’s disease were treated with nootropics and/or acetylcholinesterase inhibitors (Korea, 96 %; all other regions, 69 %), and almost all were recommended a non-pharmacological treatment (Korea, 93 %; all other regions, 100 %). Detection of mild cognitive impairment due to Alzheimer’s disease was considered prompt in Korea but suboptimal in other regions (*n* = 16) owing to low disease awareness among patients. Barriers to assessment and diagnosis included delayed healthcare visits for initial assessment (*n* = 7), neuroimaging backlogs (*n* = 6), and insufficient neuropsychology resources (*n* = 13). Access to amyloid biomarker tests, including amyloid positron emission tomography, cerebrospinal fluid analysis, and blood tests, was limited in regions other than Korea.

**Conclusions:**

The survey findings showed that screening and diagnostic processes for mild cognitive impairment due to Alzheimer’s disease in Asia require further optimization. Efforts should also be made to educate patients and caregivers, improve the diagnostic capabilities of primary and secondary healthcare providers, and reinforce cognitive screening services. The provision and reimbursement of confirmatory tests of amyloid burden should be expanded across the region to facilitate access to innovative disease-modifying therapies.

## 1 Introduction

Alzheimer’s disease (AD) represents a growing global health concern. The prevalence of AD and other dementias increased by 160.8 % from 1990 to 2019, and the fastest upward trends in age-standardized prevalence rates (ASPRs) over this period were seen in East Asia and high-income Asia Pacific countries (defined as Brunei, Japan, Korea, and Singapore) [1]. Population aging and growth are expected to contribute to further increases in the coming decades, with total prevalent cases of dementia, of which AD is the most common type, projected to grow from 57.4 million worldwide in 2019 to 152.8 million in 2050 [2]. ASPRs are set to grow by 6.2 % and 4.0 % in South Asia and East Asia, respectively (excluding the high-income Asia Pacific region) [2].

AD is a neurodegenerative disease defined pathologically according to the presence of amyloid β (Aβ) deposition, aggregation of hyperphosphorylated tau in neurofibrillary tangles (NFTs), and neurodegeneration [3]. Tests for biomarkers of these pathological hallmarks, previously used primarily in research settings, have recently become more accessible as diagnostic tools in clinical practice [3,4]. These include amyloid positron emission tomography (PET) and markers of Aβ, phosphorylated tau (p-tau), neurodegeneration/neuronal injury (neurofilament light chain [NfL]), and inflammation (glial fibrillary acidic protein) in the cerebrospinal fluid (CSF) and plasma [[3], [4], [5], [6]].

As outlined in the updated Alzheimer’s Association (AA) criteria for AD diagnosis and staging, AD begins with the appearance of disease-specific biomarkers in the absence of symptoms, referred to as Core 1 biomarkers [3]. These map onto either the Aβ or AD tauopathy pathway, and include amyloid PET, or CSF or plasma markers of Aβ42, p-tau217, p-tau181, and p-tau231. Core 1 biomarkers indicate the presence of AD neuropathologic change generally, becoming abnormal early in the course of the disease, and a diagnosis of AD can be made based on abnormal results for any one of amyloid PET, plasma p-tau217 or the ratio of p-tau217/non-phosphorylated-tau217, or CSF measures of the hybrid ratios p-tau181/Aβ42, t-tau/Aβ42, or Aβ42/40. The revised AA criteria additionally include Core 2 biomarkers (fluid biomarkers and tau PET), which become abnormal later in the disease continuum and are closely linked with symptom onset. When combined with Core 1 biomarkers, Core 2 biomarkers may be used to stage disease severity [3].

The biological entity of AD accounts for 60–80 % of all cases of dementia, a clinical syndrome characterized by progressive deficits in cognition (including memory and language) [7] of sufficient magnitude to interfere with daily function [8]. In patients with confirmed AD biomarkers, the AA criteria for AD diagnosis and staging described six clinical stages of AD, depending on the presence or absence and severity of the dementia syndrome [3]. These encompass the asymptomatic stage or preclinical AD, i.e. cognitively unimpaired (Stage 1) and transitional decline, characterized by mild detectable change with minimal functional impact (Stage 2); cognitive impairment with early functional impact (Stage 3), which corresponds to mild cognitive impairment (MCI), describing those with an objective impairment in cognition not normal for age but that does not meet dementia criteria; and AD dementia, describing patients with cognitive impairments that impact daily function and which is further subdivided into mild, moderate, and severe stages (Stages 4–6) [3].

The preclinical AD phase is estimated to take place over two decades [9], beginning with Aβ deposition, followed by NFTs and neurodegeneration, before eventually manifesting in clinical symptoms of dementia [10]. Early diagnosis and intervention (e.g. with Aβ-targeted disease-modifying therapies [DMTs]) are critical to disrupting downstream pathological processes and slowing or preventing cognitive decline [10].

It is posited that treatment may be ineffective beyond a certain, yet unestablished neuropathological threshold [6]. Indeed, neurodegeneration downstream of Aβ deposition is thought to have already occurred by the time a patient presents with MCI [11]. However, intervention at this stage may still bring clinically meaningful benefits, as demonstrated by large Phase III clinical trials of Aβ-targeting monoclonal antibodies such as lecanemab and donanemab, in which progression on clinical measures of cognitive and functional decline in patients with MCI due to AD and mild AD dementia was slower in the lecanemab and donanemab groups than in the placebo arms [12,13].

With the availability of new DMTs, healthcare systems need to optimize care pathways to ensure that suitable candidates can benefit. In Korea, a National Dementia Plan has been implemented since 2008 and is now in its fourth iteration [14]. As part of this agenda, the National Responsibility Policy for Dementia Care was announced in 2017 [15], under which the government committed to providing up to 90 % health insurance coverage for dementia-related medical expenses, opening dementia support centers in 256 regions, and expanding the provision of long-term care services [15,16]. Moreover, the public Long-Term Care Insurance scheme for the elderly was extended in 2014 to cover dementia care, and again in 2018 to provide ‘cognitive assistance’ for people with dementia, including access to day-care-center benefits and family respite care [17]. However, while Korea has made strides in providing for patients with dementia, a recent study nonetheless found that the small number of dementia specialists in the country is likely to impact its capacity to introduce new DMTs [18]. While Indonesia [19], Malaysia, Singapore, Thailand, and Taiwan [20] also have dementia plans in place, other regions in Asia are lagging. As such, challenges for dementia care remain in Asia, particularly in the assessment and diagnosis of patients with MCI due to AD.

In consideration of the changing treatment landscape, the Asia PRIME project was designed to characterize the current care pathway for MCI due to AD in Asia, including challenges and gaps in assessment and diagnosis, and to anticipate the future care landscape. We describe the results of this qualitative study and make recommendations for optimizing the care pathway for MCI due to AD in readiness for the availability of anti-amyloid DMTs in Asia.

## 2 Methods

The Asia PRIME project employed a purposive sampling strategy to recruit neurologists, geriatricians, and psychiatrists actively involved in the diagnosis and management of MCI due to AD. Physicians from the specialties working across general/tertiary referral centers and specialist memory clinics in major urban centers in Hong Kong, India, Indonesia, Korea, Malaysia, the Philippines, Singapore, Taiwan, and Thailand were invited to participate in a semi-structured interview. The selection criteria comprised having ≥10 years of clinical experience in dementia care and involvement in the management of ≥10 patients with MCI due to AD per month.

Physicians from mainland China and Japan were not included in this study as country-specific preparedness studies already exist.

Interviews were 60–90 min in length and conducted by teleconference between October 7, 2022 and October 11, 2023.

A discussion guide was developed (see Supplementary Material), comprising 34 questions organized into five topics:

1. Patient presentation and the care pathway
2. Assessment, diagnosis, and communication to the patient
3. Treatment (pharmacological and non-pharmacological)
4. Management and follow-up
5. The future landscape of AD treatment

Responses were manually reviewed and thematically analyzed. Qualitative responses to questions on these themes were framed into statements and were presented alongside the number and/or proportion of physicians, in either all or specific regions of Asia, whose answers supported these statements. Where respondents were asked to provide numerical data or estimates, the data were presented using descriptive statistics.

For cognitive screening tools, neuropsychological tests, dementia severity rating tools, psychiatric measures, neuroimaging, fluid biomarkers, and genetic risk factors, physicians were asked to indicate which tests they used and whether they were essential or optional as part of the diagnostic workup. In addition to considering responses from all participants, responses from physicians in Korea were also compared with those of physicians from other regions of Asia, given the large number of respondents from Korea and the relatively advanced state of dementia care in the country relative to other regions.

Based on the findings of the Asia PRIME project and the authors’ clinical experience, recommendations were provided for optimizing the assessment and diagnosis of MCI due to AD and preparing healthcare systems for the availability of anti-amyloid DMTs in Asia.

## 3 Results

In total, 44 physicians were interviewed. Over half practiced in Korea (*n* = 24; 54.5 %), with most being neurologists (*n* = 31; 70.5 %). Demographic data are presented in Table 1.

**Table 1:** Demographic data for interview respondents (physicians).

Respondent characteristics	Value
**Number of respondents,*****n*****(%)**	44 (100)
**Region,*****n*****(%)**	
Hong Kong	2 (4.5)
India	3 (6.8)
Indonesia	2 (4.5)
Malaysia	2 (4.5)
Philippines	3 (6.8)
Singapore	2 (4.5)
Korea	24 (54.5)
Taiwan	3 (6.8)
Thailand	3 (6.8)
**Specialty,*****n*****(%)**	
Neurology	31 (70.5)
Psychiatry	11 (25.0)
Neurology and psychiatry	1 (2.3)
Geriatrics	1 (2.3)
**MCI patients treated per month,*****n***	
Minimum	3
Maximum	400
Median	67.5
Range of proportion with MCI due to AD, %	24.0–87.5

AD, Alzheimer’s disease; MCI, mild cognitive impairment.

Physicians managed a median of 67.5 patients with MCI per month, but the range was wide (3–400). The proportion of patients with MCI due to AD ranged from 24.0 % to 87.5 %.

### 3.1 Patient presentation and care pathway

Between 21 % and 24 % of patients had initially sought opinions from other healthcare professionals and brought previous test results to their consultations. Rating the trustworthiness of such previous test results on a Likert scale of 1 (not at all trustworthy) to 5 (very trustworthy), physicians most frequently selected 4 (*n* = 19). However, some physicians noted that structural brain imaging tests were less trustworthy because of no or unspecialized review by radiologists (*n* = 3; e.g. no specific commentary on medial temporal lobe [MTL] atrophy or patterns of focal atrophy), or because of low quality/having too few slices (*n* = 3).

Most physicians noted that their patients’ chief complaint was memory loss (*n* = 42). They also reported that patients frequently presented with other symptoms, including anxiety, depression, and aggressive behavior (*n* = 8).

### 3.2 Assessment and testing

When taking a patient’s history, most physicians took into consideration cognitive function (by domain of impairment; *n* = 39), conducted neurological examinations (*n* = 37), and assessed whether symptoms impacted activities of daily living (*n* = 30). Behavioral and psychological symptoms were investigated by one-third of physicians (*n* = 13), and time of symptom onset by one-fifth (*n* = 9).

Brief neuropsychological examinations were primarily conducted after taking the patient’s history (Table 2). Most physicians reported using the Mini-Mental State Examination (*n* = 41) and largely considered it to be essential. Fewer physicians (*n* = 29) used the Montreal Cognitive Assessment, which was largely considered optional in Korea; those in other regions considered it essential (*n* = 17).

**Table 2:** Tests employed during the diagnostic workup and the proportion of physicians who considered them to be essential or optional.

	Total (*n* = 44)			Korea (*n* = 24)			Other Asia regions (*n* = 20)		
	*n* (%)	Essential, *n* (%)	Optional, *n* (%)	*n* (%)	Essential, *n* (%)	Optional, *n* (%)	*n* (%)	Essential, *n* (%)	Optional, *n* (%)
**Cognitive screening tools**									
MMSE	41 (93.2)	39 (88.6)	2 (4.5)	24 (100.0)	24 (100.0)	0	17 (85.0)	15 (75.0)	2 (10.0)
MoCA	29 (65.9)	19 (43.2)	10 (22.7)	10 (41.7)	2 (8.3)	8 (33.3)	19 (95.0)	17 (85.0)	2 (10.0)
KDSQ	14 (31.8)	11 (25.0)	3 (6.8)	14 (58.3)	11 (45.8)	3 (12.5)	0	0	0
**Neuropsychological tests**									
SNSB	19 (43.2)	19 (43.2)	0	19 (79.2)	19 (79.2)	0	0	0	0
CERAD	10 (22.7)	7 (15.9)	3 (6.8)	6 (25.0)	5 (20.8)	1 (4.2)	4 (20.0)	2 (10.0)	2 (10.0)
ADAS-Cog*	17 (38.6)	9 (20.5)	8 (18.2)	7 (29.2)	0	7 (29.2)	10 (50.0)	9 (45.0)	1 (5.0)
**Dementia severity rating tools**									
CDR	40 (90.9)	39 (88.6)	1 (2.3)	24 (100.0)	24 (100.0)	0	16 (80.0)	15 (75.0)	1 (5.0)
GDS	30 (68.2)	28 (63.6)	2 (4.5)	24 (100.0)	23 (95.8)	1 (4.2)	6 (30.0)	5 (25.0)	1 (5.0)
**Psychiatric measures**									
GDpS	31 (70.5)	29 (65.9)	2 (4.5)	24 (100)	24 (100)	0	7 (35.0)	5 (25.0)	2 (10.0)
CGA-NPI	25 (56.8)	24 (54.5)	1 (2.3)	18 (75.0)	17 (70.8)	1 (4.2)	7 (35.0)	7 (35.0)	0
**Neuroimaging**									
MRI	44 (100.0)	40 (90.9)	4 (9.1)	24 (100)	23 (95.8)	1 (4.2)	20 (100)	17 (85.0)	3 (15.0)
MRA	30 (68.2)	12 (27.3)	18 (40.9)	21 (87.5)	8 (33.3)	13 (54.2)	9 (45.0)	4 (20.0)	5 (25.0)
CT	26 (59.1)	6 (13.6)	20 (45.5)	17 (70.8)	1 (4.2)	16 (66.6)	9 (45.0)	5 (25.0)	4 (20.0)
*PET*									
Amyloid PET	34 (77.3)	4 (9.1)	30 (68.2)	23 (95.8)	2 (8.3)	21 (87.5)	11 (55.0)	2 (10.0)	9 (45.0)
^18^F-FDG PET	23 (52.3)	2 (4.5)	21 (47.7)	15 (62.5)	0	15 (62.5)	8 (40.0)	2 (10.0)	6 (30.0)
^18^F-FP-CIT-PET	20 (45.5)	0	20 (45.5)	17 (70.8)	0	17 (70.8)	3 (15.0)	0	3 (15.0)
SPECT	11 (25.0)	0	11 (25.0)	7 (29.2)	0	7 (29.2)	4 (20.0)	0	4 (20.0)
**Fluid biomarkers**									
*Blood/plasma*									
OAβ	11 (25.0)	1 (2.3)	10 (22.7)	10 (41.7)	1 (4.2)	9 (37.5)	1 (5.0)	0	1 (5.0)
Plasma Aβ	3 (6.8)	0	3 (6.8)	0	0	0	3 (15.0)	0	3 (15.0)
p-tau	3 (6.8)	0	3 (6.8)	0	0	0	3 (15.0)	0	3 (15.0)
CSF	13 (29.5)	0	13 (29.5)	9 (37.5)	0	9 (37.5)	4 (20.0)	0	4 (20.0)
**Genetic risk factors**									
*APOE* genotyping	34 (77.3)	25 (56.8)	9 (20.5)	24 (100)	21 (87.5)	3 (12.5)	10 (50.0)	4 (20.0)	6 (30.0)

⁎ Used in clinical trials only in tertiary general hospitals in Korea. Aβ, amyloid β; ADAS-Cog, Alzheimer’s Disease Assessment Scale – Cognitive Subscale; *APOE*, apolipoprotein E; CDR, Clinical Dementia Rating; CERAD, Consortium to Establish a Registry for Alzheimer’s Disease; CGA-NPI, Caregiver-Administered Neuropsychiatric Inventory; CSF, cerebrospinal fluid; CT, computed tomography; ^18^FDG, ^18^fluorodeoxyglucose; ^18^F-FP-CIT, ^18^F-N-(3-fluoropropyl)-2β-carboxymethoxy-3β-(4-iodophenyl) nortropane; GDpS, Geriatric Depression Scale; GDS, Global Deterioration Scale; KDSQ, Korean Dementia Screening Questionnaire; MMSE, Mini-Mental State Examination; MoCA, Montreal Cognitive Assessment; MRA, magnetic resonance angiography; MRI, magnetic resonance imaging; OAβ, oligomeric β-amyloid; PET, positron emission tomography; p-tau, phosphorylated tau; SNSB, Seoul Neuropsychological Test Battery; SPECT, single-photon emission computed tomography.

Comprehensive screening batteries were largely used in Korea and seldom employed in other regions. These included the Seoul Neuropsychological Screening Battery (*n* = 19; used only in Korea, where it was considered essential) and the Consortium to Establish a Registry for Alzheimer’s Disease neuropsychological battery (total, *n* = 10; Korea, *n* = 6; other regions, *n* = 4). Most physicians assessed dementia severity with the Clinical Dementia Rating (CDR; *n* = 40), which was considered an essential assessment (*n* = 39). A smaller proportion used the Global Deterioration Scale (*n* = 30), driven by physicians in Korea (*n* = 24; *n* = 6 for all other regions).

**Fig. 1:**
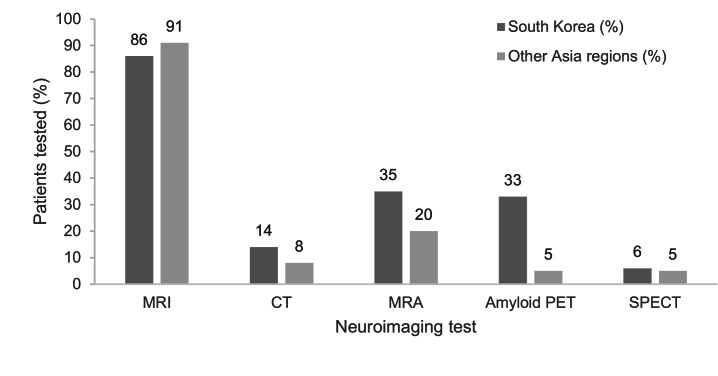
Proportion of patients undergoing each type of neuroimaging test in Korea and other Asia regions.

CT, computed tomography; MRA, magnetic resonance angiography; MRI, magnetic resonance imaging; PET, positron emission tomography; SPECT, single-photon emission computed tomography.

Neuroimaging was used by most physicians to investigate suspected MCI due to AD. The proportion of patients who received each type of neuroimaging test in Korea versus other regions in Asia is shown in Fig. 1. Magnetic resonance imaging (MRI) was ordered for most patients (Korea, 86 %; all other regions, 91 %). Almost all physicians (*n* = 40) considered MRI an essential part of the diagnostic workup, with the remaining (*n* = 4) considering it optional (Table 2). Other neuroimaging methods were primarily considered optional. Almost 60 % of respondents reported using computed tomography (CT; *n* = 26, where *n* = 17 for Korea and *n* = 9 for all other regions), which, in Korea, was considered optional (16 out of 17) and suitable for those contraindicated to MRI. While CT was deemed essential by more than half of the physicians who used it in other regions (5 out of 9), in practice, few patients were ordered CT scans (8 %; Fig. 1).

More physicians from Korea than from other regions considered amyloid PET to be essential (total, *n* = 4; Korea, *n* = 2; other regions, *n* = 2); 33 % of patients undergo an amyloid PET scan in Korea, compared with 5 % in the other regions (Fig. 1). However, physicians reported a lower rate of amyloid positivity in Korea than in all other regions (53 % vs 70 % of amyloid PET scans). Physicians in Korea reported that amyloid PET was primarily ordered for patients with atypical presentation (*n* = 5), including early onset of symptoms (*n* = 4) and unexplained or unusually fast progression (*n* = 2).

Fluid biomarkers were seldom used in Asia. Blood-based measurement of plasma Aβ oligomerization tendency (total, *n* = 11) was largely confined to Korea (*n* = 10), where it was considered optional (*n* = 9). While almost 30 % of physicians reported having used CSF tests (*n* = 13), this was always considered optional. In Korea, just 1 % of patients underwent lumbar puncture in clinical practice, while it was rarely conducted in other regions.

Over three-quarters of physicians investigated apolipoprotein E (*APOE*) genotype (*n* = 34). All physicians in Korea carried out this test (*n* = 24), most of whom considered it an essential part of the diagnostic workup (*n* = 21). Physicians outside of Korea who conducted *APOE* testing (*n* = 10) mostly considered it optional (*n* = 6).

### 3.3 Diagnosis and patient communication

Across Asia, the time taken to diagnose MCI ranged from 1 to 6 months. The average time taken in Korea was 2.8 months, but it could be up to 6 months, depending on neuropsychological testing requirements.

One-quarter of physicians (*n* = 11), all from regions other than Korea, stated that stratifying MCI according to severity was necessary or helpful. In Korea, MCI was rarely stratified by severity because there were no clear clinical criteria and MCI severity would not impact the treatment plan; half of the Korean respondents stated that stratification by disease severity was unnecessary or only required in research (*n* = 12).

When communicating the diagnosis to patients, almost all physicians explained MCI as an intermediate stage between normal aging and dementia (*n* = 42). They explained that while patients with MCI had a higher chance of progression to dementia, this was not inevitable. The remaining two doctors explained MCI as a cognitive impairment/memory problem without reference to dementia to avoid misunderstanding and causing anxiety.

### 3.4 Treatment

All physicians (*n* = 44) agreed that the goal of treatment was to slow down MCI due to AD in its progression to AD dementia. This conflicted with their patients’ expectations of a cure in most cases.

More patients in Korea were estimated to be treated with medication than in other regions in Asia (weighted percentage: 96 % and 69 %, respectively; Table 3). Weighted percentages were calculated, including two outliers for Korea (treatment rates estimated at ≤10 %) and four outliers for all other regions (treatment rates estimated at ≤30 %). Patients in all regions were treated with nootropics and/or acetylcholinesterase inhibitors (AChEIs), although treatment patterns varied. Choline alfoscerate (alpha-glycerylphosphorylcholine) was the nootropic prescribed most in Korea (71 % of patients); it was ginkgo biloba in other regions (40 %). Patterns of AChEI treatment, on the other hand, were similar. Donepezil was prescribed most in both Korea (53 %) and other regions (34 %), followed by rivastigmine and galantamine (Table 3). All physicians noted that medication was prescribed for the duration of the patient’s life.

**Table 3:** Pharmacological and non-pharmacological treatments prescribed for patients diagnosed with MCI due to AD in Asia.

	Proportion (weighted %) of patients treated	
	Korea (%)	Other Asia regions (%)
**Pharmacological treatments**	96	69
*Nootropics*		
Choline alfoscerate	71	7
Ginkgo biloba	17	40
Nicergoline	2	13
Oxiracetam	3	0
Piracetam	0	8
Souvenaid	0	2
*Acetylcholinesterase inhibitors*		
Donepezil	53	34
Rivastigmine	10	10
Galantamine	3	1
**Non-pharmacological treatments**	93	100
*Lifestyle modification*		
Continuous social and cerebral activity	95	100
Exercise	96	100
Diet	64	72
Alcohol and smoking cessation	74	69
*Psychological interventions*		
Cognitive interventions	66	61
Psychotherapy	17	25

AD, Alzheimer’s disease; MCI, mild cognitive impairment.

Almost all patients with MCI due to AD were prescribed some form of non-pharmacological intervention (Korea, 93 %; all other regions, 100 %; Table 3). The most widely recommended non-pharmacological interventions were lifestyle modifications. Exercise and continuous cerebral and social activities were recommended for 95–100 % of patients, while diet and alcohol/smoking cessation were recommended for 64–74 % of patients. Small proportions of patients were prescribed psychological interventions. Cognitive interventions were recommended for 61–66 % of patients, while psychotherapy was recommended for only up to 25 %.

### 3.5 Patient follow-up

Follow-up visits were scheduled every 3–6 months. Most physicians (*n* = 43) felt that the current interval was appropriate, given the typically slow progression of AD.

Repeat neuropsychological testing was conducted regularly, ranging from every 3–6 months (in Hong Kong, India, Indonesia, the Philippines, and Thailand) to every year (in Korea, the Philippines, and Taiwan). MRI was repeated less frequently, typically when patients presented for follow-up with rapidly progressing symptoms that could raise suspicion of another condition (e.g. stroke). Physicians in Korea reported that MRI may be repeated every 2–5 years to monitor progression. However, repeat amyloid PET was not common in Korea because of its cost; as such, few physicians reported repeating amyloid PET every 2–5 years (*n* = 2).

### 3.6 Barriers in the patient pathway

While physicians in Korea indicated that MCI due to AD was detected promptly, detection was suboptimal according to physicians in other Asian regions (*n* = 16), driven by low disease awareness among patients.

Patients across Asia faced delays in diagnosis owing to difficulties in obtaining hospital appointments or tests. Physicians in Hong Kong, India, Malaysia, the Philippines, and Thailand reported delays in initial hospital visits (*n* = 7), while backlogs in neuroimaging were reported in Malaysia, Indonesia, and Thailand (*n* = 5). In Korea, delays in diagnosis were driven by limited availability of and long waiting periods for neuropsychological testing (*n* = 11). Physicians in Taiwan noted a lack of manpower for conducting neuropsychological tests (*n* = 2).

Access to AD biomarker tests differed for patients across Asia. In Korea, amyloid PET was readily available but expensive; hence, physicians noted that it should be reimbursed (*n* = 6). Amyloid PET was not available in India, Indonesia, or Malaysia (*n* = 7). CSF biomarker testing was rarely conducted in Asia, in part because it was not available in the clinical setting (Hong Kong, Indonesia, Malaysia, the Philippines, Singapore, Taiwan; *n* = 14), but also because of its invasive nature. Two physicians in Korea noted that amyloid PET was preferred over CSF testing for biomarkers of amyloid accumulation. Blood biomarkers of AD pathology were actively used in the Philippines (*n* = 3). However, these tests were not readily available in other regions of Asia, and use remained limited. Such tests were only recently introduced at selected centers in Korea (*n* = 9) and Taiwan (*n* = 2).

Challenges in the treatment, consultation, and follow-up of patients were noted by physicians across Asia. In Korea, there was a lack of patient and caregiver education during the treatment and consultation process (*n* = 15). Physicians noted that while such education would help patients understand the disease and empower them to implement lifestyle modifications, it was limited by time and cost constraints. In other regions, adherence to recommended non-pharmacological interventions was poor (*n* = 7).

### 3.7 Expected future landscape of treatment for MCI due to AD

Following the launch of amyloid-targeted DMTs, physicians expected that there would be a greater need for amyloid biomarker tests (*n* = 35), including amyloid PET, blood, and CSF tests. Physicians in Korea emphasized the importance of treating patients with MCI due to AD as early as possible to delay progression to AD dementia (*n* = 22), while all physicians from other regions agreed that such DMTs would be helpful in treating patients with MCI due to AD (*n* = 20).

## 4 Discussion

This study illuminates the medical journey of patients with MCI due to AD in Asia, including variations in the experiences of patients and physicians across the region, the barriers faced, and physicians’ expectations following the availability of anti-amyloid DMTs.

Previous studies conducted in other regions have evaluated the preparedness of healthcare systems and infrastructure and identified barriers to the prompt diagnosis of patients with MCI due to AD. In the USA, a simulation analysis showed that projected capacity was insufficient for handling the expected caseload and predicted that patients with MCI would have to wait an average of 18.6 months for treatment, with approximately 2.1 million patients developing AD dementia between 2020 and 2040 while on waiting lists. Addressing capacity constraints was deemed challenging as it required solving a labyrinth of issues regarding payment policy, regulatory requirements, workforce considerations, and capacity planning at national and local levels, combined with awareness campaigns [21].

A study in Europe also highlighted the limited capacity to deliver care for patients with MCI due to AD. In six EU countries, peak waiting times for patients with MCI due to AD ranged from 5 months for treatment in Germany to 19 months for evaluation in France. Specialist capacity was the rate-limiting factor in France, the UK, and Spain, with treatment-delivery capacity being an issue in most countries. When DMTs become available, the projected capacity constraints could result in over 1 million patients with MCI having progression to AD dementia while on waiting lists between 2020 and 2044 [22]. This study concluded that a combination of reimbursement, regulatory, and workforce planning policies, as well as innovation in diagnosis and treatment delivery, was needed to expand capacity to treat patients with MCI due to AD.

In Japan, where 4500 people had received an anti-amyloid DMT as of November 2024 [23], a trial studying perceptions of people with preclinical AD and clinical specialists showed that patient prioritization for DMTs in the context of resource constraints and other limitations is key. While participants noted a high acceptance rate for *APOE* testing for risk assessment, this test lacked support from local guidelines and is currently not covered by health insurance in Japan [24]. In China, lecanemab is approved for patients with early AD, but considerable gaps in healthcare system preparedness were identified that impair timely AD treatment access, with the hospital-centered and episodic model of care delivery resulting in lengthy waiting times [25].

Furthermore, studies of the major barriers to diagnosing MCI due to AD in developed economies such as the USA and EU have emphasized issues similar to those reported in this study. These included patients seeing cognitive decline as a normal part of aging and not disclosing symptoms, long waiting lists or inadequate time for evaluating patients, and limited treatment options and definitive biomarker tests [26].

The present study also reaffirmed many of the barriers to prompt diagnosis described in prior studies and gave some measure of the relative impact of these barriers from the perspective of physicians from different regions of Asia. In many regions of Asia included in this study, physicians reported that detection and diagnosis of MCI due to AD was suboptimal, with the exception of Korea, where patients were diagnosed promptly. This may reflect the long-running implementation of a National Dementia Plan in Korea, which has been updated four times since it was first implemented in 2008 [14], the fourth iteration being concerned with the early diagnosis and treatment of AD [27]. Such policies have been successful in expanding reimbursement for dementia care, leading to reduced out-of-pocket costs for patients with dementia [16] and the expansion of Korea’s Long-Term Care Insurance to cover dementia management in 2014 [27].

In other regions in Asia, limiting factors in the diagnosis of MCI due to AD were access to assessments (resulting from insufficient numbers of dementia specialists), amyloid PET scans, CSF tests, and neuropsychology services. Patient discomfort with the invasiveness of CSF tests, as well as the high cost and radiation burden associated with amyloid PET, may have limited the use of these tests. Moreover, the cost of tests may impact physicians’ choices of tests, particularly for amyloid PET, with physicians across Asia noting its high cost. While MRI was broadly used across Asia for the diagnosis of MCI due to AD, variability in its reimbursement across Asia should be considered in terms of its potential impact on access to DMTs because of the need to monitor for amyloid-related imaging abnormality (ARIA) among patients treated with anti-amyloid antibodies. ARIA-H (hemosiderin deposition), in particular, cannot be monitored using CT, instead requiring a long-echo-time (T2*-weighted) gradient echo or other susceptibility-weighted sequences [28]. This may pose challenges for the implementation of anti-amyloid DMTs in regions such as Hong Kong, the Philippines, Thailand, and Taiwan, where MRI either is not reimbursed at all for patients with MCI during follow-up (Hong Kong and the Philippines) or is reimbursed only if indicated by a change in symptoms (Thailand and Taiwan).

Facilities for *APOE* genotyping were also readily available and affordable in Korea, supporting its routine use in the diagnostic workup. Conversely, *APOE* genotyping was rarely employed in the other regions of Asia included in this study, although this may not be related to the accessibility or affordability of testing. Rather, physicians may refrain from carrying out such tests because they may cause unnecessary worry for the patient, require genetic counseling, and ultimately are not imperative for a diagnosis of MCI due to AD. However, physician attitudes to *APOE* genotyping may change following the advent of amyloid-targeting DMTs in Asia, when the safe use of such drugs will be important because of the greater risk of ARIA among *APOE4* carriers [28].

The barriers to early diagnosis and assessment of MCI due to AD highlighted in the present study are aligned with other studies on the preparedness of healthcare systems for implementing anti-amyloid DMTs [[29], [30], [31]]. A literature review and survey of regional experts across the Western Pacific reported broad variability in the readiness of healthcare systems for using anti-amyloid therapies in MCI and early dementia due to AD [31]. In particular, many regions were found to lack the necessary workforce and infrastructure to monitor patients for ARIA [31]. Similar conclusions were drawn in Canada, where a review found that the healthcare system was not ready for DMTs as it cannot accommodate the need for early diagnosis, biomarker testing, and MRI monitoring [29]. A separate working group of experts in Asia [30] described a best practice framework for institutional readiness for anti-amyloid DMTs based on five pillars: clinical evaluation and diagnostic modalities; infusion protocols and infrastructure; monitoring, identification, and management of ARIA and AEs; cross-disciplinary collaboration; and education. Within the framework, cross-disciplinary collaboration can help to address resource constraints where the development of new infrastructure (e.g. dedicated centers for assessment or infusion centers for treatment delivery) is not feasible. Moreover, education of patients, carers, and physicians of various specialties underpins all other best practices described. Similar recommendations have been made for the Canadian healthcare system [29,32].

### 4.1 Expert recommendations

With the advent of DMTs, it is imperative to streamline the processes for screening and diagnosing MCI due to AD to ensure that patients can receive timely treatment, and to ensure the preparedness of healthcare systems to accommodate these treatments. Table 4 outlines recommendations for optimizing assessments of possible MCI due to AD and for preparing healthcare systems for their availability. Based on the findings of this study and the considerations outlined above, broad recommendations can also be made for improving detection and follow-up in Asia:

• Patients, caregivers, and the public should be educated about MCI and AD, including the benefits of early detection, as well as about brain health, dementia risk factors, and the importance of lifestyle modification. Such education would help reduce the stigma and bias associated with cognitive decline. Patient and caregiver education may be facilitated by longer consultations and chargeable codes for patient education services. Public information campaigns would also serve to raise awareness of MCI and prompt a more proactive approach to healthcare visits for patients.
• To tackle challenges in delayed access to diagnostic tests, primary and secondary hospitals should be strengthened in their capabilities to detect MCI due to AD early and make appropriate assessments and diagnoses. This should be supported by educating healthcare professionals, including primary care physicians (PCPs), allied health professionals, and support staff, on MCI due to AD, including biomarkers of AD pathology, the associated tests, and available treatments. Recent proposals to optimize the care pathway and facilitate early diagnosis could help bridge the gap between primary and secondary/specialist care by dividing the diagnostic workup and care pathway between PCPs and specialists into first-line (symptom detection and assessment) and second-line (biomarker confirmation, treatment, and monitoring) processes, respectively [33]. At the secondary/tertiary care level, expertise in neuroimaging analysis should also be enhanced, including education on the need for capturing sufficient slices on MRI to permit the assessment of MTL atrophy. Automating or delegating tasks in the evaluation process would also help to strengthen capacity [21].
• Patients presenting for examination should first be screened with rapid dementia staging tools for assessing cognitive and functional impairment, such as the Quick Dementia Rating System [34]. Those for whom such tests suggest a CDR score indicative of MCI due to AD or mild dementia should be referred for comprehensive neuropsychological assessment. In line with previous proposals outlined above [33], such assessments could be coordinated at the primary care level. As further support, neuropsychological and cognitive assessment services should be enhanced to create greater capacity for neuropsychological testing and to standardize the interpretation of test results. To achieve this, more clinical neuropsychologists should be trained, allowing the administration of comprehensive test batteries and providing detailed test results for physicians’ consideration. Furthermore, risk stratification based on neuropsychological screening tests and other factors could help triage and prioritize patients at increased risk of disease progression [21].
• Improvements in access to and use of biomarker testing are needed to support accurate diagnosis of MCI due to AD. Real-world evidence is also needed to standardize and support the use of blood-based biomarker tests, which are relatively affordable and simple to conduct and can help alleviate resource constraints. Guidelines and medical education for healthcare professionals are needed to support the clinical use of blood-based biomarkers once they become available (no such tests are approved by the US Food and Drug Administration at the time of writing).
• Wider provision of amyloid PET facilities across Asia and appropriate reimbursement will also be needed. Careful and strategic planning of infrastructure will be necessary. The projected reduced need for amyloid PET in the mid- to long-term once other biomarker tests become more widely available must also be considered. Broader access to biomarker testing also requires the approval, commercialization, and reimbursement of other recently developed innovative diagnostics (e.g. plasma Aβ, plasma p-tau, tau-PET) in healthcare systems across Asia.
• To ensure the appropriate and cost-effective implementation of biomarker testing, expert consensus on a biomarker algorithm and best practices should be developed, including treatment plans for patients with MCI due to other etiologies. Furthermore, there should be worldwide harmonization of screening and diagnostic methods using biomarkers, helping to define risks and devise novel approaches for the prevention of AD dementia.
• Drawing from the appropriate use recommendations (AUR) of the Alzheimer’s Disease and Related Disorders Therapeutics Work Group [35], AUR for anti-amyloid DMTs should be developed at the country or regional level once they become available, driven by local or regional medical societies to ensure deep insight into and alignment with national healthcare system infrastructures, available resources, and reimbursement environments.

**Table 4:** Expert recommendations for optimizing assessments of possible MCI due to AD and ensuring healthcare system preparedness for implementing anti-amyloid DMTs.

**Optimizing assessment and diagnosis of MCI due to AD** • All patients aged ≥60 years presenting with a cognitive complaint (including those raised by a care partner) should undergo: full evaluation, including medical history capturing comorbidities, medications, etc.; assessment with cognitive and behavioral screening tools; blood tests to exclude other conditions affecting cognition (e.g. hypothyroidism, vitamin B12 deficiency, anemia, etc.); and brain imaging. • Patients aged ≥60 years presenting/diagnosed with depression, anxiety, and/or psychosis should also be routinely screened for cognitive and behavioral impairments suggestive of AD. • Evaluation of patients with cognitive complaints should comprise an assessment of the patient’s mood, activities of daily living, and cognition. Clinicians should select structured screening tools with demonstrated sensitivity and specificity for detecting MCI or mild AD and that they are comfortable administering in everyday practice. Where cognitive screening indicates impairment, this should be followed up with a full neuropsychological evaluation to accurately characterize cognitive deficits and interpret test results. • A complete MRI protocol should be implemented for patients aged ≥60 years with cognitive complaints. If MRI is not available/feasible, CT should be conducted instead. MRI protocols should include at least T1-weighted structural imaging with comprehensive coverage of the MTL; T2-weighted FLAIR can be considered to exclude vascular disease, diffusion-weighted imaging to exclude prion disease, and SWI to exclude hemorrhagic lesions and cerebral amyloid angiopathy. The minimum inclusion of T2-weighted gradient-echo imaging with or without SWI sequences should be mandated in MRI protocols once anti-amyloid DMTs become available to enable safe treatment initiation and monitoring of treatment-related ARIAs. • For a confirmatory diagnosis of MCI due to AD, patients should be referred to a dementia specialist, who should confirm a diagnosis based on clinical findings and the presence of Aβ, investigated using amyloid PET or CSF testing, whichever is available/feasible. • Where blood biomarkers are available or approved in the future, these should be used to triage screening or as confirmatory tests if validated as adequately sensitive and specific for Aβ accumulation. • Following a diagnosis of MCI due to AD, a treatment and management plan should be made, incorporating available and appropriate pharmacological treatments and non-pharmacological approaches/lifestyle modifications where necessary, ensuring adequate social support, assessment of capacity to make personal and financial decisions and plans for designating an alternative decision-maker in case of loss of capacity, and, if driving, monitoring of driving safety. Patients who are AD biomarker negative should be followed up to determine the etiology of their MCI and a care plan arranged as appropriate. **Ensuring healthcare system preparedness for implementing anti-amyloid DMTsI. Patient identification and selection** • Optimize screening, assessment, and diagnostic processes to identify patients with early-stage AD or other amyloid-related disorders and streamline referral processes. • Implement and streamline biomarker testing (amyloid PET, CSF, and blood-based biomarkers) to guide treatment decisions. • Establish criteria for selecting patients most likely to benefit from anti-amyloid DMTs. **II. Treatment management** • Develop and implement standardized treatment protocols for anti-amyloid DMTs, including dosing and administration. • Develop strategies to monitor treatment response, including cognitive and functional assessments. • Establish systems for monitoring potential side effects (ARIAs). **III. Infrastructure and resources** • Establish dedicated dementia clinics or centers of excellence. • Educate HCPs on anti-amyloid DMTs, including patient selection, treatment monitoring, and AE management. **IV. Patient education and support** • Educate patients and caregivers on anti-amyloid DMTs, including benefits, caveats, and potential risks. • Offer support services to help patients and caregivers manage the complexities of anti-amyloid DMTs. **V. Collaboration and communication** • Foster collaboration among HCPs, including neurologists, psychiatrists, geriatricians, emergency physicians, and PCPs, to ensure comprehensive care. • Encourage patient and caregiver shared decision-making and support patient-centered care. • Establish effective communication channels among healthcare providers, payers, and policy-makers to ensure equitable access to treatment. **VI. Research and quality improvement** • Establish registries and systems for collecting and analyzing real-world data on anti-amyloid DMTs, including effectiveness, safety, and patient outcomes.

Aβ, amyloid β; AD, Alzheimer’s disease; AE, adverse event; ARIA, amyloid-related imaging abnormality; CSF, cerebrospinal fluid; CT, computed tomography; DMT, disease-modifying therapy; FLAIR, fluid-attenuated inversion recovery; HCP, healthcare professional; MCI, mild cognitive impairment; MRI, magnetic resonance imaging; MTL, medial temporal lobe; PCP, primary care physician; PET, positron emission tomography; SWI, susceptibility-weighted imaging.

### 4.2 Study limitations

This study described patterns in and barriers to the assessment, diagnosis, and treatment of MCI due to AD as perceived by physicians. Variability in how physicians interpreted the questions or provided responses could have introduced bias and affected data consistency, limiting the reliability of some findings. Additionally, self-reported responses may have been influenced by recall bias or social desirability. As such, the study should not be interpreted as an objective measure of the situation in Asia but, rather, a subjective narrative collected from physicians. While the response rate was adequate, it may not fully represent the diversity of clinical practices across specialties, potentially affecting the generalizability of the results. Furthermore, many of the barriers included in this study could be investigated in significantly greater depth; qualitative research to explore these themes in further detail might help reveal more about the underlying factors.

Participants in this study included neurologists, geriatricians, and psychiatrists. Given that a multitude of healthcare professionals manage patients with MCI due to AD in the real world, the group of physicians participating in this study may not reflect the overall clinical situation. The degree to which the sample represents the relevant sectors of the clinical community should be considered when interpreting the results and drawing conclusions. Moreover, as the study did not survey physicians in all regions of Asia (e.g. physicians from mainland China and Japan were not included), generalizability of the results to the wider Asia region may be limited.

Finally, the study also did not include other stakeholders such as patients, family members, and caregivers, who will have different perceptions about current practices and barriers. Involving this wider audience will be an important avenue for future research to gain a holistic overview of the current disease landscape.

## 5 Conclusions

This study described clinical practices for assessing and diagnosing MCI due to AD in Asia and evaluated barriers faced by patients. Screening and diagnostic processes in Asia should be optimized to improve the detection of MCI due to AD and reduce the time to diagnosis. Efforts should also be made to educate patients and caregivers, improve the diagnostic capabilities of primary and secondary healthcare providers, and reinforce capacity in cognitive screening services. Finally, the provision and reimbursement of confirmatory tests of amyloid burden should be expanded across Asia to facilitate access for patients to new, innovative amyloid-targeting treatments.

## Funding

The work described here was supported by Eisai Co., Ltd, Tokyo, Japan.

## Ethical standards

The authors adhered to the publication ethics specified by the *Journal of Prevention of Alzheimer’s Disease*. This is an original work, and all authors have contributed importantly to its creation. The authors have verified the accuracy of the content of the article.

## CRediT authorship contribution statement

**Seong Hye Choi:** Writing – review & editing, Writing – original draft. **SangYun Kim:** Writing – review & editing, Writing – original draft, Validation. **Paulus Anam Ong:** Writing – review & editing, Writing – original draft. **Ai Vyrn Chin:** Writing – review & editing, Writing – original draft. **Jacqueline Dominguez:** Writing – review & editing, Writing – original draft. **Christopher Li-Hsian Chen:** Writing – review & editing, Writing – original draft. **Vorapun Senanarong:** Writing – review & editing, Writing – original draft. **Chaur-Jong Hu:** Writing – review & editing, Writing – original draft. **Manjari Tripathi:** Writing – review & editing, Writing – original draft. **Vincent Mok:** Writing – review & editing, Writing – original draft. **Gandan Jiang:** Writing – review & editing, Writing – original draft, Project administration, Data curation, Conceptualization. **Amitabh Dash:** Writing – review & editing, Writing – original draft, Project administration, Data curation, Conceptualization.

## Declaration of competing interest

The authors declare the following financial interests/personal relationships which may be considered as potential competing interests:

SangYun Kim reports administrative support and statistical analysis were provided by IQVIA. Ai Vyrn Chin has received speaker's honoraria from Lundbeck, Menarini, and Eisai, and sponsorship for accommodation and conference attendance from Eisai within 36 months prior to the publication of this manuscript. All other authors declare that they have no known competing financial interests or personal relationships that could have appeared to influence the work reported in this paper.

## Appendix Supplementary materials

Image, application 1
