# Supplementary material for: The patient pathway for mild cognitive impairment due to Alzheimer’s disease in Asia: Current practices, barriers, and expert recommendations for optimization
Source: J Prev Alzheimers Dis. 2025 Jun 6;12(7):100215. doi: 10.1016/j.tjpad.2025.100215 (PMC12321631; doi:10.1016/j.tjpad.2025.100215)
Supplement: Supplementary file 1 [file mmc1.docx]

**Asia PRIME Project for MCI**

Questionnaire Guideline (V3.5, ENG)

Aug 2023

| **ID** |  |
| --- | --- |
| **Hospital** |  |
| **Name** |  |
| **Specialty** |  |
| **Interview date** | **. . .** |

| **Part 0.** | **Introduction** |
| --- | --- |

- **Introduction: A brief introduction about the company and the moderator**
- **Topic: Identify the overall process of AD MCI management and advise on optimal patient care directions in the future**
- **Time: Approximately 60-90 minutes**
- **Guide: Information about personal information protection and recording**

Hello, my name is OOO, and I work as a consultant. Thank you for taking the time out of your busy schedule to speak with me.

Throughout the interview, we will discuss the process of patient management for AD MCI, as known as MCI due to Alzheimer’s disease. Going forward, we will use the term AD MCI to refer to this condition. Please note that when we discuss **AD MCI**, we will refer to **patients diagnosed by a specialist's clinical decision**, **not just** those **confirmed by CSF or Amyloid PET test**.

Our discussion will cover examination, treatment, and follow-up based on your clinical experience. The ultimate goal of the interview is to establish an ASIA Expert Consensus on optimal management of AD MCI patients. Please feel free to share your opinions, as there are no correct answers to the questions we will be discussing.

During the interview, you may be asked questions regarding the number of patients or the percentage of certain items. Please keep in mind that you do not need to provide an exact numeric value, and **it is acceptable to answer based on your approximate perception**. So, feel free to share your best estimate based on your experience and knowledge.

The interview will take **approximately 60-90 minutes**, and we will be recording our conversation for analysis purposes only. **The recording will be** **kept for 5 years** and then destroyed. Your insights and opinions are highly valued and will be used solely for research and interview purposes.

**Before we dive into AD MCI, we would like to briefly gather some background information including the general number of patients and their distribution.**

**Q1.** On average, how many **patients with dementia** do you **treat per month?**
Please answer including AD (Alzheimer's disease) dementia, vascular dementia, and other types of dementia.

**Q2.** On average, how many **MCI patients** do you **treat per month?**

**Q20.** Could you please provide the **percentage breakdown of MCI patients** you are currently treating, **categorized by etiology** **[Total=100%]**

| **Self-completion Q1, Q2, Q20** |
| --- |

**
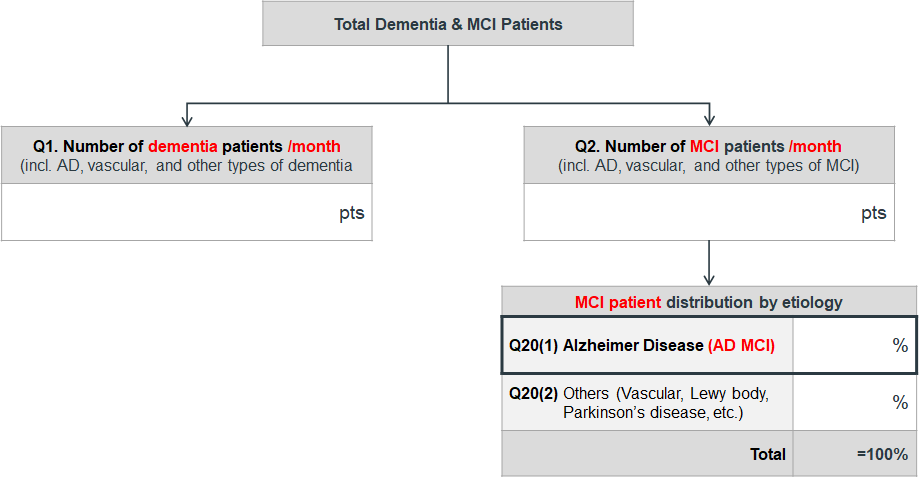
**

**Q0-2.** Now, we would like to ask you about **national insurance coverage or subsidies for treating Alzheimer's Disease** in [YOUR COUNTRY].

**[Moderator: Questions for each country / If the respondent finds it difficult to answer the questions, skip them.]**

**[Indonesia]**

(1) In the case of Indonesia, majority of the population receive healthcare benefits through the National Health Security System, JKN (Jaminan Kesehatan Nasional).

a. Does JKN provide coverage for AD MCI and AD dementia?
**[Probing: Check if the national insurance coverage for MCI / dementia is different]**

**[Malaysia]**

(1) The medical system in Malaysia can be divided into two sectors: the public sector and the private sector.

a. Does the National Health Insurance Scheme (NHIS), which is part of the public sector, provide coverage for AD MCI and AD dementia?

**[Probing: Check if the national insurance coverage for MCI / dementia is different]**

**[Philippines]**

(1) In Philippines, most of the population receive healthcare benefits through the National Health Insurance Program (NHIP).

a. During the Pioneer Meeting, we observed that **national health insurance does not provide coverage for AD MCI**. If this is accurate, we would like to confirm if this also applies to AD dementia.

**[Probing: Check if the national insurance coverage for MCI / dementia is different]**

**[Singapore]**

(1) In the case of Singapore, it is known that dementia treatment payments can be withdrawn through a Medisave account.

a. Does Medisave also provide coverage for AD MCI which is not dementia?

**[Thailand]**

(1) The medical system in Thailand can be divided into two sectors: the public sector and the private sector.

a. Does public health insurance provide coverage for AD MCI and AD dementia?

**[Probing: Check if the national insurance coverage for MCI / dementia is different]**

**[Taiwan]**

(1) In Taiwan, the government shares medical expenses through the NHI (National Health Insurance).

a. Does NHIP provide coverage for AD MCI and AD dementia?

**[Probing: Check if the national insurance coverage for MCI / dementia is different]**

**[India]**

(1) The medical system in India can be combined to two sectors: the public sector and the private sector.

a. Do national healthcare scheme, like Ayushman Bharat, or subsidies provide coverage for AD MCI and AD dementia?

**[Probing: Check if the national insurance coverage for MCI / dementia is different]**

**[Hong Kong]**

(1) The medical system in Hong Kong has a dual-track system: the public sector and the private sector. And public hospitals offer affordable and subsidized healthcare.

a. Are AD MCI and AD dementia also covered by subsidies or reimbursed?

**[Probing: Check if the national insurance coverage for MCI / dementia is different]**

**As a final background question, we would like to ask about medical treatment conditions.**

1. **How many patients** do you typically **treat in a day**?

(1) On average, how long does it take you to treat patients?
a. Then, how long does it take you to treat AD MCI patients on average?

b. When treating AD MCI patients, is there a difference in treatment hours between new patients and returning patients?

| **Part B.** | **Assessment & Diagnosis** |
| --- | --- |

**We will now proceed to ask detailed questions step by step, covering the entire process of patient management from the ‘diagnosis of MCI to follow-up.’**

**From now on, I will ask you about the process of diagnosis of AD MCI.**

**Q12.** Regarding patients who are currently being treated for AD MCI, what do you typically **ask during their initial visit?** Specifically, what **aspects of the patient's history must be considered** when making a diagnosis of MCI?
**[Probing point: Listen to medical history from a patient/caregiver, check patient's family history, check patient/caregiver's complaints about memory/cognitive impairment, etc.]**

**Q7.** Regarding the AD MCI patients currently under treatment, what is the proportion of patients who came directly to the current hospital for MCI, compared to those who had previously visited other hospital/institution? Please provide your approximate estimate.

(1) If patients have visited other hospitals before coming to see you, **what types of hospitals** did they usually visit?

- Primary hospital
- Secondary hospital
- Tertiary or General hospital

a. Did they receive **drug prescriptions** or other treatments **at least once** before?

**Q13.** Among the AD MCI patients who have visited other hospital previously, what is the proportion of **patients who bring their prior test results from a previous hospital**, compared to patients without any test results? Please provide your approximate estimate.

| **Q7. Whether to visit other hospital/institution** | | **Patient proportion** | |  |
| --- | --- | --- | --- | --- |
| 1 | Patients who came to the current hospital directly | % | |  |
| 2 | Patients who have visited another hospital/institution previously | % | |  |
| **Total** | | **=100%** | |  |
| **↓** | | **↓** | |  |
| **Q13. Whether to bring their prior test results** | | | | |
| 1 | Patients who brought their prior test results  from previous hospital | | % | |
| 2 | Patients who came to the hospital without any test results  from previous hospital | | % | |
| **Total** | | | **=100%** | |

**[Moderator: Ask if Q13 >0%]**

(1) If patient visit with test results from the previous hospital, **what test results do they usually bring?**

a. How much do you **trust the test results from the previous hospital**? Please rate your trust level on a scale of 1 to 5, where 1 means you don't trust the results at all and 5 means you trust them completely.

| **No trust at all** | | **Neutral** | | | **Very trusting** | |
| --- | --- | --- | --- | --- | --- | --- |
| 1 | 2 | | 3 | 4 | | 5 |

- Then, does the credibility of the test results depend on the hospital that conducted the test?

**Q22-1.** Regardless of examination, **what is your criteria** to make the final decision **to diagnose AD MCI?**

**[Probing: certain neuropsychological test or brain imaging test result, etc.]**

| **Test Type**  ★ : The questions in the Pre-survey are marked with the corresponding symbol. | | | **Q14(1)** | **Q14(2)** | **Q16(1)** | |
| --- | --- | --- | --- | --- | --- | --- |
|  |  |  | Essential | Additional | Hospital routine O | Hospital routine X |
| Neuro-  psychological test | | MMSE (Mini-Mental Status Examination) ★ | 1 | 1 | 1 | 2 |
|  |  | HDS (Hasegawa Dementia Scale) | 2 | 2 | 1 | 2 |
|  |  | MoCA (Montreal Cognitive Assessment) ★ | 3 | 3 | 1 | 2 |
|  |  | SMCQ (Subjective Memory Complaints Questionnaire) | 4 | 4 | 1 | 2 |
|  |  | ADAS-cog (Alzheimer's Disease Assessment Scale-Cognitive subscale) ★ | 6 | 6 | 1 | 2 |
|  |  | CDR (Clinical Dementia Rating) ★ | 7 | 7 | 1 | 2 |
|  |  | GDS (Global Deterioration Scale) ★ | 8 | 8 | 1 | 2 |
|  |  | QDRS (Quick Dementia Rating System) | 47 | 47 | 1 | 2 |
|  |  | ADL (Activities of Daily Living) | 9 | 9 | 1 | 2 |
|  |  | DSQ (Dementia Screening Questionnaire) | 10 | 10 | 1 | 2 |
|  |  | GDpS (Geriatric Depression Scale) | 11 | 11 | 1 | 2 |
|  |  | CERAD (Consortium to Establish a Registry for Alzheimer’s Disease) ★ | 13 | 13 | 1 | 2 |
|  |  | LICA (Literacy Independent Cognitive Assessment) | 14 | 14 | 1 | 2 |
|  |  | CGA-NPI (Caregiver-Administered Neuropsychiatric Inventory) | 39 | 39 | 1 | 2 |
|  |  | IADL (Instrumental Activities of Daily Living) | 40 | 40 | 1 | 2 |
|  |  | Other Neuropsychological test: Specify- |  |  |  | |
| Brain imaging test | | MRI★ | 15 | 15 |  | |
|  |  | MRA | 16 | 16 |  | |
|  |  | CT★ | 17 | 17 |  | |
|  |  | Amyloid-PET★ | 18 | 18 |  | |
|  |  | FDG PET | 41 | 41 |  | |
|  |  | CIT-PET (dopamine PET) | 42 | 42 |  | |
|  |  | SPECT | 20 | 20 |  | |
|  |  | Other Brain imaging test: Specify- |  |  |  | |
| Gene test | | Apolipoprotein E (apolipoprotein E Genotype) ★ | 21 | 21 |  | |
|  |  | Other Gene test: Specify- |  |  |  | |
|  | | CBC test★ | 43 | 43 |  | |
| Lab test | | Urine test★ | 22 | 22 |  | |
|  |  | Stool test | 23 | 23 |  | |
|  |  | Chest X-ray | 24 | 24 |  | |
|  |  | Electroencephalography (EEG) | 25 | 25 |  | |
|  |  | Electrocardiogram (ECG or EKG) | 26 | 26 |  | |
|  |  | Liver function test | 27 | 27 |  | |
|  |  | Kidney function test | 28 | 28 |  | |
|  |  | Thyroid function test | 29 | 29 |  | |
|  |  | Electrolyte test | 30 | 30 |  | |
|  |  | Blood sugar test | 31 | 31 |  | |
|  |  | Lipid test | 32 | 32 |  | |
|  |  | VDRL (Venereal Disease Research Laboratory) | 33 | 33 |  | |
|  |  | AIDS test(HIV Test) | 34 | 34 |  | |
|  |  | Vitamin test: Vitamin B12 | 35 | 35 |  | |
|  |  | Vitamin test: Vitamin D | 48 | 48 |  | |
|  |  | Other Vitamin test: Specify |  |  |  | |
|  |  | Homocysteine test | 44 | 44 |  | |
|  |  | Other lab test: Specify - |  |  |  | |
| Cerebrospinal fluid (CSF) test★ | | | 36 | 36 |  | |
| Blood  Marker | Oligomerized Amyloid Beta, OAβ | | 37 | 37 |  | |
|  | Plasma Amyloid Beta, pAβ | | 45 | 45 |  | |
|  | Plasma Tau, pTau | | 46 | 46 |  | |
|  | Other blood marker test: Specify | |  |  |  | |
| Neurological test  (Evaluation of various neurological functions such as sensation, atrophy of motor nerves or muscles, walking ability, and reflexes) | | | 38 | 38 |  | |
| Other test: Specify | | |  |  |  | |

**[Moderator: Ask questions using showcards]**

**Q14.** Could you describe the **examination process used in hospitals to diagnose AD MCI?**

- Before we begin, please note that the following questions are focused **solely on the clinical practice settings** and not on clinical research settings.
- Provide a **step-by-step account of the process**, including the order in which each test is conducted.
- Please tell us in detail **who is involved in each test**. If there is a **separate team or staff** responsible for conducting diagnostic tests, please provide more information about that.

(1) Which test is considered **essential for diagnosing AD MCI**?

a. **When do you typically conduct** these essential tests after a patient’s first visit?

b. **Who conducts** each test? (Doctor oneself vs. Resident vs. Other Speciality or staff)

c. Is it possible to **differentiate between MCI and dementia with essential tests alone**?

d. **What kind of patients** do you think **require additional tests?**

(2) **What additional tests** are conducted beyond the essential tests **by the patients?**

(3) **Which type of patients** do you conduct each test on?

a. Does it vary **depending on the type or cause of suspected cognitive impairment**? If so, please explain how it will change.

b. **When** are these additional tests usually performed after the essential examination?

c. Who **conducts** each additional test? (Doctor oneself vs. Resident vs. Other Speciality or staff)

**Q16.** I would like to inquire about **neuropsychological tests** you conduct.

(1) How long does it take to conduct the neuropsychological tests in your hospital, from the initial visit to the actual testing?

(2) Are the neuropsychological tests that you conduct **commonly used in other hospitals in [YOUR COUNTRY]?**

(3) If so, are the neuropsychological tests that you are required to perform commonly conducted in the hospital you are working? Do you have any other **essential tests** that you conduct that is **not part of the hospital routine**?

a. If so, what tests are they?

b. What is the reason for conducting these tests?

**Q17.** Next, I would like to ask you more information on the **brain imaging tests**.

(1) Considering your current AD MCI patients as 100%, **what percentage** of the patients receive each type of brain imaging test? **[Each brain imaging test ≤100%]** Please tell us **based on the brain imaging test** that you conduct **to diagnose AD MCI.**

**.**

| **Percentage of each brain imaging test** | | **Patient ratio** |
| --- | --- | --- |
| 15 | MRI | % |
| 16 | MRA | % |
| 17 | CT | % |
| 18 | Amyloid-PET | % |
| 20 | SPECT | % |
|  | Other Brain imaging test: Specify- | % |

**[Moderator]: Check the pre-survey responses in advance and ask questions]**

**a. (Ask questions for each test that has not been conducted at all)** Is the brain imaging test available but not conducted in the hospital, or is the brain imaging test not available at all in the hospital? ★

**[Moderator: Ask if Q17(1)(18) Amyloid-PET >0%]**

(2) Considering AD MCI patients who received Amyloid-PET scan as 100%, what percentage of your patients tested **positive for amyloid-beta** (Aβ) above the threshold level? **[≤100%]**

| **Amyloid-PET test result** | **Ratio** |
| --- | --- |
| Amyloid-beta (Aβ) positive | % |

a. **For which type of patient** do you conduct an Amyloid-PET test?

b. If the **accumulation of amyloid-beta** is **already clear without the test,** would you decide **not to conduct** Amyloid-PET test?

**[Moderator: Ask if answered Q14 (36) CSF (Cerebrospinal fluid)]**

**(3)** Considering AD MCI patients whom you are currently treating as 100%, what percentage of your patients receive a **CSF test**? **[≤100%]**

a. In which case do you conduct the CSF test? What is the reason for conducting a CSF test?

**[Moderator: Ask only if there is any response on Q14 Blood Biomarker]**

(4) I would like to ask about the blood biomarker test, which is used to collect blood samples from potential AD MCI patients and measure **beta-amyloid or Tau protein accumulation**.

a. **How often** do you conduct blood biomarker test? **[≤100%]**
**[Moderator: If more than one tests are conducted, ask by each test]**

b. **In what case** do you conduct blood biomarker test?
- Then, what is the reason for conducting the biomarker test in that case?

**c. How much** do you **trust the blood biomarker testing result**? Please rate your trust level on a scale of 1 to 5, where 1 means you don't trust the results at all and 5 means you trust them completely.

| **No trust at all** | | **Neutral** | | | **Very trusting** | |
| --- | --- | --- | --- | --- | --- | --- |
| 1 | 2 | | 3 | 4 | | 5 |

**[Moderator: Ask only if there is no response on Q14 Blood Biomarker]**

d. I noticed that you don’t typically conduct blood biomarker test. Can you tell me if **there are any blood biomarker test available in [YOUR COUNTRY]** associated with cognitive function?

e. If so, what is your **reason for not conducting the test** even though it is available?

**Q19.** Is it **necessary to classify patients with AD MCI by severity** after examination, and what is the reason for doing so?

(1) If you classify patients by severity, what criteria do you use to determine the severity level? **[Probing point: CDR score, amnestic or non-amnestic, etc.]**

(2) If patients are classified according to their level of severity, how does the treatment plan vary across different severity levels?

**Q21.** On average, **how long does it take** for AD MCI patients to **receive that diagnosis** following their initial visit with you? (From the first visit ~ to MCI judgment)

year month

(1) Regarding the period you mentioned, do you think there has been a delay in diagnosis? If so, is there a specific reason behind the delay**? [Probing: backlog of tests or administrational issue]**

**O3.** Do you think the detection of AD MCI patients is currently well-executed in [YOUR COUNTRY]?

(1) What factors may **make it difficult to detect AD MCI** patients?

(2) In your opinion, **should** patients with cognitive impairment be **detected earlier**, **at least at the MCI level**?

**Q22-2.** If there are any ambiguities or areas that require improvement in the **AD** **MCI diagnosis,** what would they be?

(1) What tests do you believe are **necessary for an appropriate diagnosis** of AD MCI in the future, **even if they are not currently being conducted**? Why do you believe these tests are important?

a. Why is it currently not possible to conduct these tests?

b. What kind of support would be necessary to implement these tests in the hospital setting?

c. In your opinion, which tests do you think should be supported by the government?

**Q23.** Then, **what changes in the** **diagnosis itself or criteria** do you expect after anti-amyloid treatment launch?

**I would like to ask more about how you communicate with patients and caregivers at diagnosis.**

**Q24. Who usually explains AD MCI** to the patient and their caregiver(s) at diagnosis?

**(Doctor oneself/ Resident/ Nurse/ other staff, etc.)**

**Q25.** When explaining 'AD MCI' to patients and caregivers, what specific information do you provide? Please **describe the language you use** as specifically as possible.

(1) Do you **use the term 'MCI'** when explaining the condition to patients and caregivers?

(2) Do you explain the **relationship between MCI and dementia?**

(3) **What topics do you typically cover** when explaining to patients and caregivers? **[Probing: Check if it includes the following]**

- Reasons for conducting each test
- Test results (specific figures)
- Future process (prognosis)
- Treatment goal
- Treatment method and process

(4) Are there any obstacles to overcome or areas that need improvement in terms of how AD MCI is explained to patients and caregivers? **[Probing point: Education materials, person in charge of MCI education, etc.]**

**Q27**. After explaining at the diagnosis of AD MCI, does your hospital offer any additional **education programs for patients or caregivers**?

**[Moderator: Ask (1)~(3) if education program is offered]**

**(1) What topics are typically covered** in **patient** education programs?

**a. Where, and how** does the education work? (1:1 training, group training, etc.)

**b. Who are the** **primary educators** involved in patient education**?** (Doctor oneself, Resident, Nurse, Others)

**c. How often** is patient education conducted? (One-time, once a month, once every three months, once every six months, once a year, etc.)

**d.** On average, **how much time** is **spent on patient education**?
(less than 30 minutes, 30 minutes to 1 hour, 1 hour to 2 hours, etc.)

(2) What **topics are typically covered** in the **caregiver** education program?

**(3) Is the government support fee** set for the education? If so, what are the **patient out-of-**pocket **levels?**

**[Moderator: Ask (4) if education program is not offered]**

(4) If the education programs are **not currently available, what is the reason** for their absence?

**[Moderator: Ask all]**

(5) In your opinion, what are some **key components to deliver effective education programs** for AD MCI patients and their caregivers **in a hospital setting?**

| **Part D.** | **Management & Follow-up** |
| --- | --- |

**From now on, we would like to ask you more questions about follow-up of AD MCI patients.**

**Q33.** Usually **at what intervals do AD MCI patients make follow-up visits** to you? (Once a month, once every three months, once every six months, once a year, etc.)

(1) In your opinion, **how often** should the patients **visit for follow-up?**- If there is a difference between the actual follow-up visit frequency and the ideal frequency, what accounts for it?

**Q34.** **What follow-up tests** are typically performed for patients with AD MCI?

**[Moderator: Refer to the previous list of tests]**

(1) What tests do you believe are essential for the patients?

(2) Under what circumstances should additional tests be performed, and why?

(3) What is the optimal testing interval for each essential test and additional test?

**O19. What do you usually ask** when AD MCI patient visits **for a follow-up**?

(1) What do you particularly focus on during the consultation?

(2) When any changes are detected during the consultation, do you think additional examination is necessary?
a. What is the primary test you conduct during that time?

**O17.** Do you think **collaboration with primary/secondary hospitals is necessary to follow-up** on AD MCI patients? Please explain your reasoning.

| **Part C.** | **Treatment** |
| --- | --- |

**From now on, I would like to ask you about the treatment of AD MCI patients.**

**Q28. What treatment goals** do you have for AD MCI patients?

(1) Is there a case where the patient's treatment goal is different from you?

**Q29.** Considering **AD MCI patients whom** you are currently treating as 100,

what is the percentage (%) of **patients who are undergoing medication treatment?** (**≤100%**)

**[Ask if ratio of medication treatment <100%]**

(1) What kind of patients are currently not undergoing medication treatment?

(2) Considering AD MCI patients (Q29(1) response) who are currently undergoing medication treatment from you as 100, **what is the percentage of patients prescribed by the following regimens?**

If you have any medication prescribed through clinical trials, please answer to 'clinical trial medication' and if possible, please tell us the specific medication name.

Total sum may be greater than or equal to 100, as a patient is likely to be prescribed in combination with multiple medications. (Total ≥100%)

| **Q29. (1) Administration of drug treatment** | | | **Patient ratio** |
| --- | --- | --- | --- |
|  |  |  | % |
|  | | | ↓ |
| **Q29. (2) Ingredient name** | | | **Patient ratio** |
| Nootropics | 2 | Choline Alfoscerate | % |
|  | 3 | Nicergoline | % |
|  | 4 | Oxiracetam | % |
|  | 5 | Gingko biloba | % |
|  | 991 | Other nootropics: Specify - | % |
| AChEI | 6 | Donepezil | % |
|  | 7 | Galantamine | % |
|  | 8 | Rivastigmine | % |
|  | 992 | Other AChEI: Specify - | % |
| Others | 9 | Vitamin D | % |
|  | 997 | Other medications | % |
| **Total** | | | **≥100%** |

**(3) How long do you prescribe** patients medication for MCI due to AD (AD MCI)?

______________year months

**Many pharmaceutical companies are currently conducting clinical trials on anti-amyloid treatments. It is expected that anti-amyloid agents will be used in clinical practice settings in the near future.**

.

**O9.** If this type of anti-amyloid treatment becomes available in the future, do you think it would be **ideally helpful in treating AD MCI**?

(1) Why do you think so?

(2) When considering prescribing this treatment, **which type of patients would you prioritize**? **[Moderator: if physician comments only on economic power, please advise it can be assumed that there are no constraints on drug pricing.]**

(3) In order to prescribe anti-amyloid treatment, various tests should be conducted, such as Amyloid-PET or CSF. Do you believe that **government support for testing could affect** your **intention to prescribe** the treatment?

(4) Considering the future landscape of treating MCI due to AD, including anti-amyloid treatment, **what improvements and changes** **should be made to the national insurance system in [YOUR COUNTRY]**?

**O10.** If the anti-amyloid treatment is approved in [YOUR COUNTRY] in the future**,** do you think it would **change patient’s awareness of AD MCI?**

(1) Do you believe that the **diagnosis of AD MCI will become more active** if the anti-amyloid treatment is approved in the future?

(2) Would these changes vary **depending on insurance coverage or government support**??

**O20.** What do you think would be the **biggest obstacle** in prescribing anti-amyloid treatment? **How** could this **obstacle be overcome**?

(1) According to FDA standards, a **patient's amyloid-beta positive results must be confirmed** before prescribing anti-amyloid treatment. Considering the current circumstances, where amyloid PET devices are rare or CSF test rates are low, **what alternative methods** could be used as solutions??

a. If so, do you think it is **necessary to discuss with the relevant authorities for the**

**method to be settled** as a standard method in the future? Why do you think so?

**Q30.** From now on, I would like to ask you about **non-medication treatment**.

(1) Considering **AD MCI patients whom** you are currently treating as 100, what is the percentage (%) of **patients who are undergoing non-medication treatment?**

(2) If so, consider MCI due to AD (AD MCI) patients who are undergoing non-medication treatment (Q32(1)) as 100. **What percentage of these patients do you recommend non-medication treatment for? Total sum may be greater than or equal to 100, as a patient may be recommended multiple methods. (Total ≥100%)**

| **Q32. (1) Recommendation of non-medication treatment** | | **Patient ratio** |
| --- | --- | --- |
|  |  | % |
|  | | **↓** |
| **Recommended non-medication treatment methods** | | **Patient ratio** |
| 1 | Cognitive based (intervention) program  – Cognitive training, cognitive stimulation, cognitive rehabilitation, reality awareness training, recall | % |
| 2 | Psychotherapy | % |
| 3 | Exercise | % |
| 4 | No-smoking, No-alcohol | % |
| 5 | Sustained social and cerebral activity | % |
| 6 | Diet | % |
| 997 | Other non-medication treatments: specify | % |
| **Total** | | **≥100%** |

(3) Among the non-medication treatment methods, you have answered, are there any programs **implemented in your hospital**?

a. **(If answered yes)** Is there a set **government subsidy fee** for the program?

**O16.** What are the **general challenges or inconveniences associated with following up on patients** who are receiving treatment (medication/non-medication) for AD MCI?

(1) What kind of difficulties do **patients** complain about?

(2) On the other hand, what kind of difficulties do **caregivers** complain about?

(3) What are the plans to solve the problems you’ve mentioned above?

| **Part A.** | **Visit of Patients with AD MCI** |
| --- | --- |

**From now on, we will talk about the hospital visit process of the AD MCI patients who are currently being treated, by reviewing how they visited you at first.**

**Q5.** What was the condition of the AD MCI in their first visit?

(2) Among the various symptoms you have mentioned, what are the **main symptoms in particular that make patients decide to visit the hospital**?

a. In general, what is the proportion of **patients who first recognize their symptoms themselves**, compared to be recognized by others? Please provide your approximate estimate.

| **First person who recognized symptoms** | | **Patient proportion** |
| --- | --- | --- |
| 1 | Patients themselves (self-awareness) | % |
| 2 | By others | % |
| **Total** | | **=100%** |

b. Then, do the patients **typically come to the hospital voluntarily**, or recommended by people around them?

**Q6.** During the initial visit to the hospital, do AD MCI patients **tend to accompany others**, compared to come alone? Please provide your approximate estimate.

| **Whether to accompany family/others**  **at the first visit** | | **Patient proportion** |
| --- | --- | --- |
| 1 | Accompany others | % |
| 2 | Come alone | % |
| **Total** | | **=100%** |

**O5.** Lastly, in your opinion, **what aspects of the entire process** of visit, screening, diagnosis, treatment, and follow-up for MCI patients **require improvement**? And **how should they be improved**? Please provide suggestions that are **not limited to the hospital you work at**.

- Disease awareness education to the public
- Education about primary/secondary hospitals or non-specialized medical personnel with

cognitive disabilities

- Medical system/system improvement
- Aid/support in the community
- National policy support
- Others

**– Thank you very much for taking the time to participate in the interview. –**
